# A case of imported *Leishmania infantum* cutaneous leishmaniasis; an unusual presentation occurring 19 years after travel

**DOI:** 10.1186/s12879-014-0597-x

**Published:** 2014-11-27

**Authors:** Amy Crowe, John Slavin, Damien Stark, Craig Aboltins

**Affiliations:** Department of Infectious Diseases, St Vincent’s Hospital, Melbourne, Australia; Department of Pathology, St Vincent’s Hospital, Melbourne, Australia; Department of Microbiology, SydPath, St Vincent’s Hospital, Darlinghurst, New South Wales Australia; Department of Infectious Diseases, Northwest Academic Centre, The University of Melbourne, Epping, Victoria Australia

**Keywords:** Cutaneous leishmaniasis, Parasitology, Neglected tropical infectious diseases, Microbiology, Liposomal amphotericin

## Abstract

**Background:**

*Leishmania infantum* is a flagellated protozoan parasite that is able to parasitize blood and tissue. *Leishmania* species cause a spectrum of clinical disease with cutaneous, visceral or mucosal involvement. *L. infantum* is recognised as a cause of visceral leishmaniasis (VL) and is less commonly reported as a cause of cutaneous leishmaniasis (CL) from countries around the Mediterranean basin. This is the first report of imported *L. infantum* CL to Australia and is remarkable for a 19 year period between the patient's exposure to an endemic region, and the manifestation of symptoms.

**Case presentation:**

A 76 year old Italian-born man presented to our institution with a non-healing lesion over his upper lip, abutting his nasal mucosa. The patient had travelled to Italy, an endemic area for *L. infantum* 19 years earlier but had resided in Australia, a non-endemic area since. Histopathology performed on a biopsy of the lesion demonstrated findings consistent with CL. A species specific polymerase chain reaction (PCR) performed on the tissue detected *L. infantum*. The patient had complete clinical recovery following treatment with Liposomal amphotericin B at a dose of 3 mg/kg for five days followed by a subsequent 3 mg/kg dose at day ten.

**Conclusions:**

*L. infantum* should be recognised as a cause of imported CL in returned travellers from the Mediterranean. In this case, the incubation period for *L. infantum* CL was at least 19 years. This case adds to the described spectrum of clinical presentations of leishmaniasis and supports the theory of parasite persistence underlying natural immunity and recurrence of disease. Clinicians should consider *L. infantum* CL in the differential diagnosis of a non-healing skin lesion in any patient who reports travel to the Mediterranean, even when travel occurred several years before clinical presentation.

**Electronic supplementary material:**

The online version of this article (doi:10.1186/s12879-014-0597-x) contains supplementary material, which is available to authorized users.

## Background

*Leishmania* species are flagellated protozoa that parasitize the blood or tissue. Infection is transmitted to humans by the bite of a female *Phlebotomus* sand fly. The classical form of visceral disease, "kala-azar", is characterized by fever, anaemia and splenomegaly. Leishmaniasis is recognized by the world health organization (WHO) as a neglected tropical disease [1]. It causes significant morbidity and mortality worldwide with an estimated 12 million people infected in over 88 countries [1]. *L. infantum* is well recognized as the etiological agent of VL in southern Europe, the Middle East and North Africa [2]. CL due to *L. infantum* has only more recently been recognized. Del Giudice et al, described *L. infantum* as a cause of CL in 3 patients and 3 adults from southern France in 1998 [3]. More recently cases from Portugal and Malta have been described [4],[5]. Herein we describe the first case of imported *L. infantum* CL into Australia. This case is made even more remarkable by the 19 year period between our patient traveling to an endemic region and presenting with disease. The implication this has for our understanding of the disease pathogenesis and immunity are discussed.

## Case presentation

A 76 year-old man was referred to our hospital with a 10 month history of an enlarging plaque on the cutaneous aspect of the upper lip. He had presented six months previously with symptoms of nasal stuffiness and epistaxis. The lesion began as a small nodule.

Past medical history included type-2 diabetes mellitus, ischaemic heart disease and hypertension. The man was born in Italy. In 1952 he immigrated to Australia. He resided in the Northern Territory for seven years then had resided in the outer suburbs of Melbourne, Victoria, since. 19 years prior to presentation he had travelled back to Italy and southern France. He denied any other travel. The man could not recall any similar facial lesions in the past.

**Figure 1:**
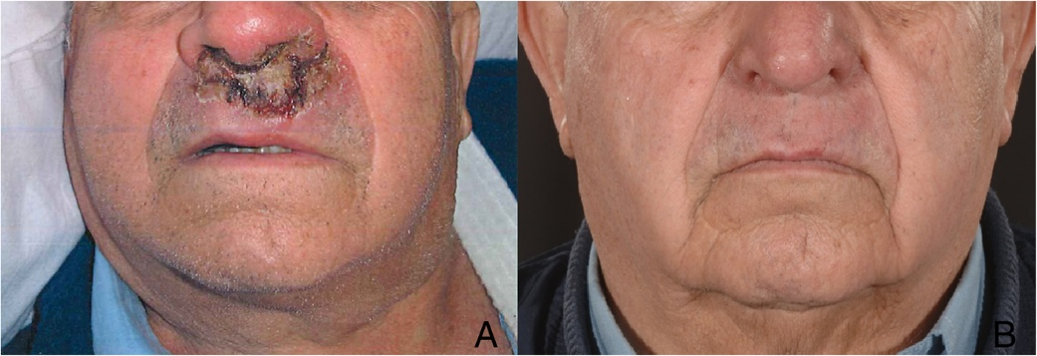
**Upper lip lesion (A) Appearance of lesion prior to treatment (B) Resolution of lesion 8 months post treatment.**

Examination revealed a 2 × 1.7 cm plaque involving the cutaneous aspect of the upper lip bordering the nostrils (Figure 1a). The lesion had a moderate exudate and some scaling. The patient was afebrile with no splenomegaly.

**Figure 2:**
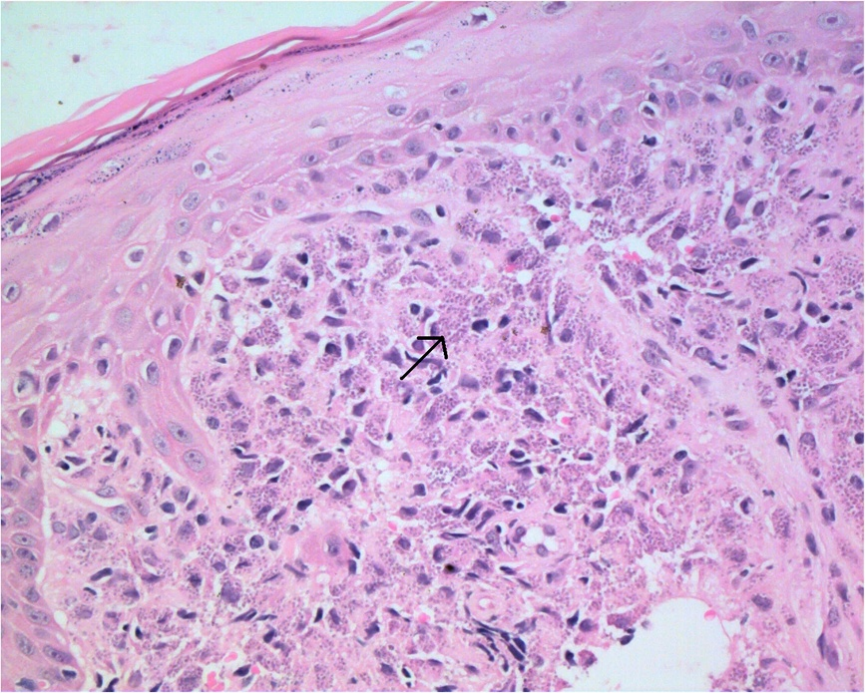
**Upper lip punch biopsy.** H&E stain (x40 magnification) demonstrating innumerable dot-shaped microorganisms suggestive of amastigote of *Leishmania* species.

Two 2 mm × 4 mm biopsies of the lesion were taken. Histopathology (Figure 2) revealed mixed suppurative and granulomatous inflammation in the dermis with prominent plasma cells. Innumerable dot-shaped microorganisms of approximately 3 microns in diameter were seen filling histiocytes. Giemsa stain of these microorganisms was positive, morphologically consistent with amastigotes of *Leishmania* species. Periodic acid Schiff and Grocott (silver) stains for fungi were negative.

The identification and speciation of *L. infantum* was confirmed by two molecular methods. Polymerase chain reaction (PCR) targeting of the internal transcribed spacer (ITS) region and subsequent digestion of the amplicon with the restriction enzyme *Hae*III was performed as previously described [6]. The restriction fragment length polymorphism (RFLP) banding pattern was consistent with *L. donovani* complex. In order to confirm genotyping results and to further speciate, the sample was analyzed by another PCR-RFLP genotyping method targeting the miniexon gene according to the genotyping scheme described elsewhere [7]. The banding pattern was consistent with *L. infantum.*

Standard bacterial cultures revealed no growth at 48 hours. A human immunodeficiency virus (HIV) antibody test was negative. Baseline renal function was within normal range. A peripheral lymphocyte count demonstrated a slight decrease in CD4 T- cells (0.48 × 10^9^/L), this persisted post treatment and no cause for this was identified.

Due to the anticipated pain of intra-lesional injections, infection with a species that causes visceral disease and concern regarding mucosal involvement, the patient was given intravenous liposomal amphotericin B (3 mg/kg) daily for five days with a further infusion at day ten. Review a month later showed minor improvement in the size of the lesion with persistent cracking and erythema. Suspecting secondary bacterial infection a course of antibiotics (Cephalexin) was prescribed. A second biopsy was performed three months post treatment. This revealed marked granulomatous and inflammatory reactions with occasional amastigotes. A further dose of liposomal amphotericin B at 3 mg/kg was given. Topical hydrocortisone was prescribed to manage surrounding inflammation. On review eight months after treatment, there was complete resolution of the lesion (Figure 1b).

## Conclusions

Leishmaniasis is distinguished by both clinical presentation and geographic origin. VL is predominantly caused by *L. donovani* complex, consisting of *L. donovani* and *L. infantum* [2]. CL is predominantly caused by *L. major*, *L. tropica*, *L. donovani* and *L. aethiopica* in the old world (Middle East, Pakistan, Africa) and by *L. mexicana*, *L. amazonensis* and *L. braziliensis* in the new world (Central and South America, Amazon basin) [2].

Post kala-azar dermal leishmaniasis, diffuse cutaneous leishmaniasis and leishmania recividans are included in the clinical spectrum of leishmaniasis [2]. Post kala-azar dermal leishmaniasis follows VL and is most commonly caused by *L. donovani* [2]. Diffuse cutaneous leishmaniasis is more commonly described in new world CL. Leishmaniasis recividans is an uncommon presentation of CL, almost exclusively caused by *L. tropica* [8]-[10] and characterized by a relapsing course and a paucity of amastigotes on histopathological examination of skin lesions [10]. Periods of up to 43 years between episodes of leishmaniasis recividans have been described [8].

*L. infantum* VL in the Mediterranean is a well described zoonosis with various Phlebotomus species of sand-fly acting as the vector and dogs serving as the primary reservoir [11],[12]. *L. infantum* causing limited cutaneous leishmaniasis (LCL) has been described in adults and children from southern France, Italy, Portugal and Malta [3]-[5]. *L. infantum* LCL mainly occurs in exposed areas and usually has a nodular appearance, although infiltrative lesions have also described [3]-[5].

Our patient did not present with symptoms until 19 years after travel to an endemic country. Previously reported cases of CL imported into Australia report onset of symptoms within months of travel to, or residence in an endemic country [13]-[15].

This case report describes a very uncommon case of *L. infantum* LCL with a very long incubation period. Reactivation of parasites in this case is likely to have occurred in the setting of declining immune function, caused by advancing age and CD4 lymphopenia. Prolonged incubation periods with old world CL have previously been described [16] and and low CD4 cell subsets have been recognized as a risk factor for recurrence in HIV-1 and *L. infantum* co-infection [17],[18]. Asymptomatic *L. infantum* parasitaemia has been documented in HIV-1 co-infected patients in endemic areas [19]. It is less likely this case represents a case of *Leishmania* recividans, as our patient has several inconsistent features; no history of preceding episodes, abundant amastigotes present on the histopathology and infection with a *Leishmania* species that has never been described to cause leishmania recividans.

A recent review of 213 cases in returned travelers identified 20 cases of old-world CL caused by *L. donovani* complex, which encompasses *L. infantum* [20]. 71% were tourists from the Mediterranean. In a series of cases imported into Australia, two were identified (by PCR) to be caused by *L. infantum*. One case presented as VL, the other presented with post-kala-azar dermal leishmaniasis. Both patients had a history of travel to Greece [21].

More recent and therefore local acquisition in our patient is unlikely. No locally acquired case of leishmaniasis in humans has ever been reported in Australia. A novel species of leishmaniasis causing CL has been described in Australian macropods (Kangaroos and Wallaroos) [22] but this species is phylogenetically distinct from *L. infantum* (the species identified by PCR in our case). Phlebotomus species of sandflies have been identified from various Australian regions (mainly Queensland) [23], so vector transmission via an imported reservoir (e.g.- infected imported dogs) is a theoretical possibility, however our patient did not report any contact with imported dogs.

Diagnosing CL in non-endemic areas is difficult and often delayed. Tissue histopathology is crucial for diagnosis. On hematoxylin and eosin stain, amastigote stages (intracellular form) can be seen within macrophages in the dermal layer. The cytoplasm of the amastogotes stains light blue, whilst the nucleus and kinetoplast (bar-shaped mitochondrial structure) stain red with Giemsa stain. Amastigotes within tissue specimens can be differentiated from fungal organisms because they do not stain positive with periodic acid-Schiff, mucicarmine, or silver stain [24].

*Leishmania* promastigotes (extracellular form) may be demonstrated from culture. Selective media such as Novy, MacNeal and Nicolle's medium are required for growth. Promastigote stages can be seen on a wet mount from media growth and stain with Giemsa [24].

Leishmaniasis serology and antigen based skin tests are problematic in non-endemic areas and are not available in Australia. Leishmaniasis PCR on tissue is sensitive and specific and allows species differentiation [6],[7],[21].

Evidence on the best management of CL caused by *L .infantum* is lacking [25]. Successful treatment with both pentavalent antimony and amphotericin has been described. We used liposomal amphotericin B with good results due to the concern about mucosal involvement and infection with a species more commonly implicated in VL.

In summary we present an unusual case of *L. infantum* CL in a 76 year old Italian man with mild CD4 lymphopenia and a history of travel to an endemic area 19 years preceding presentation. This case demonstrates that CL caused by *L. infantum* may have a long incubation period. *L. infantum* should be recognized as a cause of imported CL in patients who have travelled to the Mediterranean.

## Consent

Written informed consent was obtained from the patient for publication of this case report and any accompanying images. A copy of the written consent is available for review by the Editor of this journal.

## Authors' contributions

A/ProfessorCA conceived the paper, contributed content to the manuscript and critically reviewed the manuscript. Dr AC performed a literature review and drafted the manuscript. A/Professor JS provided the histopathology images and reviewed the manuscript for important intellectual content. Dr DS performed the molecular diagnostics and contributed information regarding this to the manuscript. All authors read and approved the final manuscript.

## Authors' information

Dr Amy Crowe is an Infectious Diseases Physician and advance trainee in Medical Microbiology currently working at Peter MacCallum Cancer Hospital, East Melbourne and St Vincent’s Hospital Melbourne, Australia. A/Professor John Slavin is an anatomical pathologist working at St Vincent’s Pathology, Melbourne, Australia. Dr Damien Stark, PhD is a senior molecular scientist working at the Department of Microbiology, SydPath, St Vincent’s Hospital, Darlinghurst, Australia. A/ProfCraig Aboltins is an Infectious Diseases Physician at St Vincent’s Hospital Melbourne and The Northern Hospital, Epping, Australia.

## Authors’ original submitted files for images

Below are the links to the authors’ original submitted files for images. Authors’ original file for figure 1 Authors’ original file for figure 2

## Abbreviations

CM: Centimetre
CL: Cutaneous leishmaniasis
LCL: Limited cutaneous leishmaniasis
HIV: Human immunodeficiency virus
KG: Kilogram
PCR: Polymerase chain reaction
RFLP: Restriction fragment length polymorphism
VL: Visceral leishmaniasis
