# Supplementary figures and images for: A case of imported Leishmania infantum cutaneous leishmaniasis; an unusual presentation occurring 19 years after travel
Source: BMC Infect Dis. 2014 Nov 27;14:597. doi: 10.1186/s12879-014-0597-x (PMC4262283; doi:10.1186/s12879-014-0597-x)

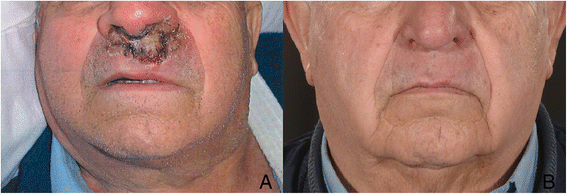

Supplement: Supplementary file 1 — Authors’ original file for figure 1 [file 12879_2014_597_MOESM1_ESM.gif]

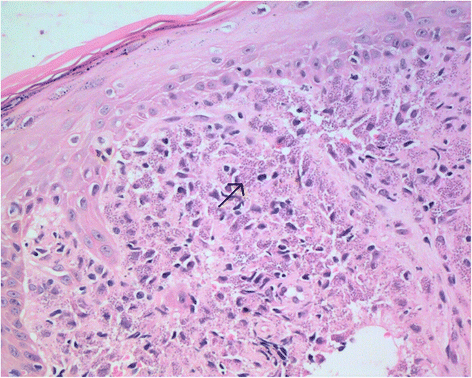

Supplement: Supplementary file 2 — Authors’ original file for figure 2 [file 12879_2014_597_MOESM2_ESM.gif]
